# The Unforeseen Impact of the COVID-19 Pandemic on Dismal Pregnancy and Fetal Outcomes

**DOI:** 10.7759/cureus.31044

**Published:** 2022-11-03

**Authors:** Jasmina Begum, Shabnam K, Pooja Sahu

**Affiliations:** 1 Obstetrics and Gynecology, All India Institute of Medical Sciences, Bhubaneswar, Bhubaneswar, IND

**Keywords:** cesarean delivery, covid-19, pregnancy, preeclampsia, pandemic, antenatal, stillbirth, maternal mortality

## Abstract

India noticed a surge in maternal mortality and stillbirth rate during the COVID-19 pandemic. This rise in mortality is not always due to viral infection by COVID-19 but is probably contributed by other determinants. We present a case of maternal mortality with stillbirth in a multigravida, at 41 weeks' gestation, with bronchial asthma, severe preeclampsia, fetal bradycardia, severe symptoms of flu infection, and a previous cesarean delivery. She delivered a stillborn baby by emergency cesarean delivery and due to adverse obstetric consequences, she succumbed. Disruption of the overwhelmed healthcare system to cope with the COVID-19 pandemic has caused unsupervised pregnancy, unprecedented delays in reaching the hospital, delays in receiving proper care, and suboptimal care due to difficulty in differentiating actual severe preeclampsia from COVID-19-induced preeclampsia-like syndrome have resulted in preventable maternal mortality and stillbirth.

## Introduction

The world is under the COVID-19 pandemic, which is caused by severe acute respiratory syndrome coronavirus 2 (SARS-CoV-2), a virus belonging to the Corona viridae family. India has noticed a surge in maternal mortality and stillbirth rate during the current COVID-19 pandemic. There is a common perception that pregnant women are prone to develop viral infections owing to hormonal and immunological changes, but it has been mentioned by health experts that the clinical course of COVID-19 pneumonia in pregnant women is the same as in non-pregnant women, so they are not considered a susceptible group [1]. In pregnant women, the case morbidity rate is up to 3% and the mortality rate is 1.2% [2,3]. A report has mentioned the effect of the COVID-19 pandemic on pregnancy outcomes is high in poorer countries and the risk of mothers dying during pregnancy or childbirth has increased by about one-third [4]. The maternal mortality ratio in Mexico increased by over 60% in one year study period during the pandemic and confirmed COVID-19 was linked to 25.4% of maternal death cases [5]. Similarly, the National Center for Health Statistics (NCHS) reported an 18.4% increase in US maternal mortality in one year and given a 16.8% increase in overall US mortality in 2020, largely attributed to the COVID-19 pandemic [6]. The rise in maternal mortality during the COVID-19 pandemic is unlikely due to viral infection solely but probably contributed by the disruption of the overwhelmed healthcare system to cope with the COVID-19 pandemic. In this study, we report a case of maternal mortality with stillbirth due to the indirect effects of the COVID-19 pandemic.

## Case presentation

An antenatal woman, (gravida 3, para 1, abortion 1) at 41 weeks of gestation was referred to us because of a lower respiratory tract infection. She presented with complaints of dry cough and breathlessness for 10 days. She had no history of fever or pain in the abdomen, leaking, or bleeding per vagina. She was a known case of bronchial asthma diagnosed 17 months prior and was on medications; however, no documents about the same were available to her. There was no history of any other chronic illness. She could not undergo regular antenatal care, nuchal translucency (NT), maternal serum markers for Down’s syndrome screening, non-invasive prenatal genetic testing (NIPT), oral glucose tolerance test (OGTT), and fetal ultrasound due to COVID-19-related lockdown restrictions nor did she attend any teleconsultation. She had undergone a cesarean section for her first pregnancy 11 years ago. On examination, she was conscious and oriented, her pulse rate was 80/min; BP - 170/130 mm of Hg; RR - 30/min, and SpO_2_ - 82% at room air which improved to 90% with 4L of O_2_. On per, abdomen examination uterus was term size with cephalic presentation. There was no scar tenderness. The fetal heart rate at admission was 76 beats/min. On per vaginum examination, the cervix was unfavorable. Her COVID-19 rapid antigen test was negative and COVID-19 RT-PCR was sent. Initial management had been done to control her blood pressure and prevent convulsions. Maximum doses of injection labetalol followed by oral nifedipine were given to control her blood pressure, oxygen inhalation, and injection of magnesium sulfate loading dose as per the Pritchard regimen was given to prevent convulsions. In spite of all these measures, her blood pressure remained on the higher side. A provisional diagnosis of G3P1L1A1 at 41 weeks with a history of previous cesarean delivery with severe preeclampsia having an acute exacerbation of bronchial asthma and HELLP syndrome or it could be a case of severe COVID-19 infection-induced preeclampsia like syndrome were made.

After initial stabilization, she was taken up for emergency lower segment cesarean section for an indication of previous cesarean delivery with severe preeclampsia, and fetal bradycardia with an unfavorable cervix, and the decision to incision time was nearly two hours. She delivered a fresh growth-restricted stillborn baby of 2,000 g. Intraoperatively the patient had placenta accrete spectrum (PAS) and atonic postpartum hemorrhage. She was given intravenous crystalloids with 40 units of oxytocin infusion and per rectal misoprostol 800 µg for atonic postpartum hemorrhage. However, she still continued to bleed, and by the end of the second hour, the patient had lost nearly two liters of blood. She developed sudden hypotension and desaturation intraoperatively for which she was transfused with 10 units of blood and blood products and the case was managed with a cesarean hysterectomy with an incision to hysterectomy duration of three hours. However, postoperatively the patient could not be extubated and she was shifted to the intensive care unit (ICU). She tested COVID-19 RTPCR negative and was on broad-spectrum antibiotics and ionotropic support for severe hypovolemic shock. Daily renal function test, liver function test, D-dimer, and coagulation profile was done which showed a worsening trend following severe hypovolemic shock and consumptive coagulopathy (Table 1).

**Table 1:** Maternal laboratory parameters

Laboratory Parameters	Reference Range	POD0	POD1	POD2	POD3	POD4	POD5	POD6	POD7	POD8
RAT for SAR-CoV-2	Positive/ negative/ inconclusive	Negative								
RTPCR, Nasopharyngeal swab positive	Positive/negative/inconclusive	Negative								
RTPCR, Bronchoalveolar Lavage	Positive/negative/inconclusive	--								Negative
Haemoglobin (g/dL)	13-17	12.2	8.6	7.0	7.3	6.6	5.2	4.4	5.7	5.9
Haematocrit	39-51	38	26.9	23.5	21.7	18.6	16.5	13.9	17.4	18.9
Total Leucocyte count*10^3/^cu-mm	4-11	8.9	16.8	14.3	16.7	10.59	7.5	7.34	12.5	22-28
Neutrophil (%)	40-80	60	87	82	85	80	79	88	80	87
Platelet count *10^3^/cu.mm	150-450	329	120	105	30-45	45-60	18	24	30	37
D-Dimer, quantitative (ug/L)	<0.5	1.5		2.39			10.1	8.82	7.1	7.62
Fibrinogen (mg/dL)	200-400			122			128			
Ferritin (ng/mL)	11-307			57.1			157		248	
PT (seconds)	11-14	19.0	23.3	34	32	22.7	23	32	33	56
APTT (seconds)	26-38	31.2	32.4	35.8	33.6	34	35	33	40	57
AST (IU/L)	5-50	335	253	261	634	6029	685	564	514	587
ALT (IU/L),	5-50	363	206	297	435	3197	793	712	736	691
Bilirubin, total (mg/dL)	0.3-1.2	0.6	0.8	1.4	1.6	1.7	2.3	3.3	3.2	3.2
Bilirubin, direct (mg/dL)	0.0-0.2	0.3	0.4	0.8	0.8	0.8	1.2	2.0	2.1	2.0
LDH (IU/L)	0-247	725		936			3876	1933		
Total Protein (g/dL)	6.7-8.6	2.0	1.8	2.0	2.1	2.8	3.5	3.8		3.1
A:G Ratio (g/dL)	0.8-2.0	1.2	1.0	1.2	1.2	1.33	1.2	1.12		1.07
Urea (mg/dL)	17-43	22	55	72	72	93	102	149		114
Creatinine (mg/dL)	0.3-1.3	0.7	1.6	2.8	2.6	4.2	4.1	5.4		6.2
Uric acid (mg/dL)	2.5-6.8	5.4	10.4	14.6	12.7	14.0	15.5	10.6		10.3
Sodium (mEq/L)	135-145	138	140	143	140	141	146	139		
Potassium (mEq/L)	3.5-5.0	3.3	4.1	4.8	4.6	5.1	4.5	4.4		5.1
Chloride (mmol/L)	102-109	95	107	112	109	112	113	111		107
Ionized Calcium (mmol/L)	1.15-1.33	0.13				0.56	O.82	0.92		0.87

**Figure 1:**
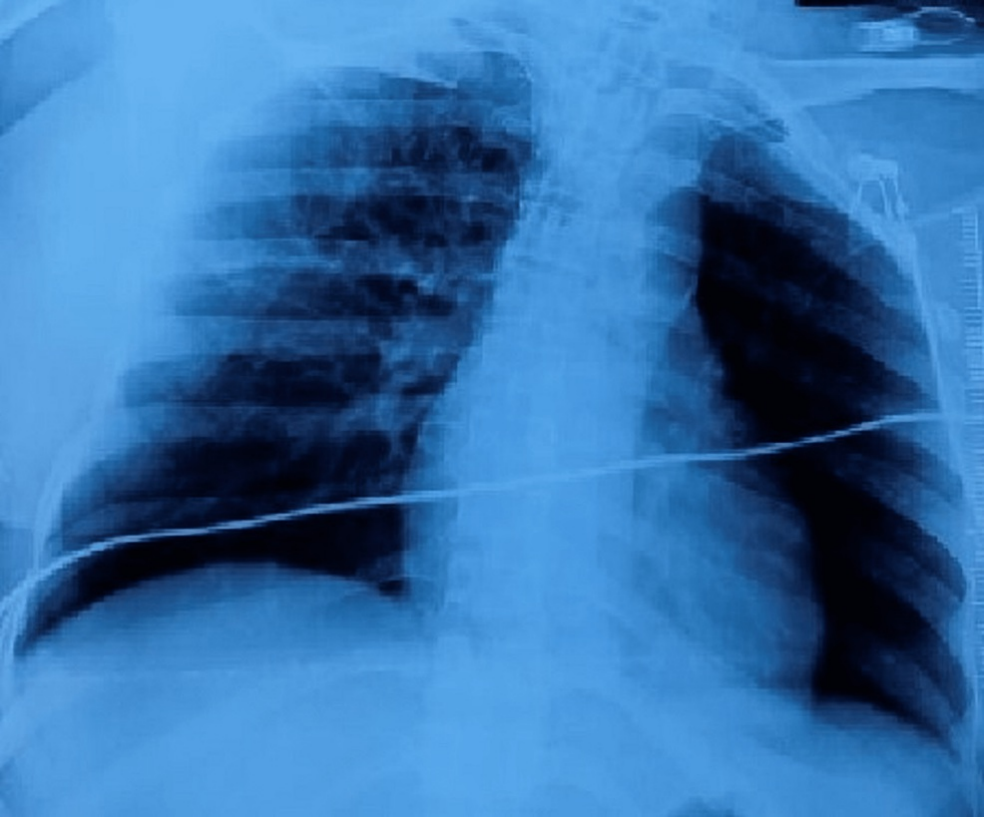
Chest x-ray showing peri-bronchial thickening, more on the right lung, increased per hilar markings, and blunting of the left costophrenic angle.

**Figure 2:**
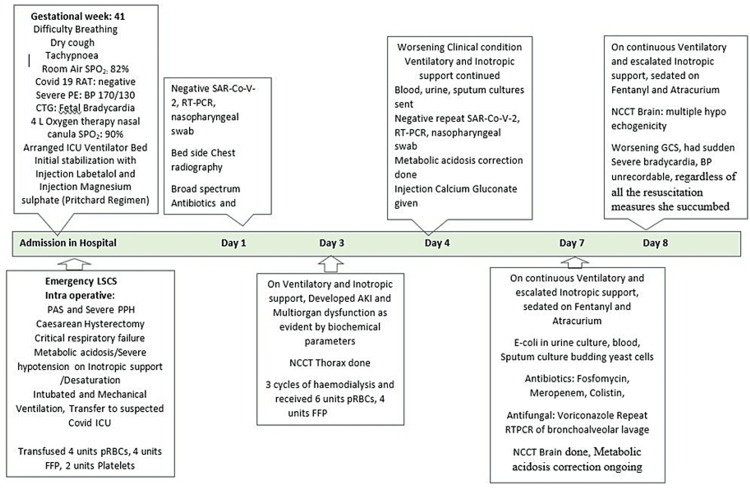
Timeline course of the disease of the patient

During her hospital course, she developed acute kidney injury with multiorgan failure and received three cycles of hemodialysis and received six units of packed cells, and four units of fresh frozen plasma during the postoperative period. Postoperative day 4, urine culture sensitivity report showed E. coli sensitivity to Fosfomycin, peripheral blood culture and sputum cultures showed budding yeast cells. Based on rising clinical suspicion, her COVID-19 sample from the nasopharynx was repeated on day 4 which was negative. A portable chest radiography for the patient was made, which revealed peri-bronchial thickening, more on the right lung, increased per hilar markings, and blunting of the left costophrenic angle was noted (Figure 1). On a postoperative day 7, she was started on broad-spectrum antifungals because of a worsening Glasgow Coma Scale (GCS) and a repeat RT-PCR of bronchoalveolar lavage was sent, which was also negative. NCCT brain showed multiple hypoechogenicity suspected to be of hypoxic injury. On postoperative day 8, she developed sudden bradycardia and her condition worsened, regardless of all the resuscitation measures she succumbed. The timeline of the course of the disease of the patient is shown in Figure 2.

## Discussion

COVID-19 is primarily a disease of the respiratory system, with 14% of cases developing viral pneumonia and 5% progressing to acute respiratory distress syndrome requiring intensive care support [7]. Severe infection of COVID-19 manifests with serious systemic effects like hypertension, thrombocytopenia, liver injury, and kidney disease [8]. Pregnant women with severe COVID-19 pneumonia are at increased risk for developing preeclampsia-like syndrome compared to the general population [8,9]. We reviewed and discussed the probable determinants that contributed to this maternal mortality and stillbirth during the pandemic. This case, presented with typical features of COVID-19 infection though the RT-PCR was thrice negative. Since the SARS-CoV-2 virus causes microangiopathy and liver injury similar to preeclampsia, a severe case of actual pre-eclampsia with HELLP syndrome can mimic a severe case of COVID-19 infection-induced preeclampsia-like syndrome thereby making the diagnosis difficult for the treating physician. Also, in a patient with underlying lung disease like bronchial asthma as in this case, a superadded pre-eclampsia with HELLP syndrome mimicked severe COVID-19 infection. This misdiagnosis, however, might have resulted in a delay in receiving prompt adequate care as she was referred from two hospitals before admission to our hospital. There was also a delay in starting cesarean delivery for fetal distress due to the need for initial stabilization of the patient, arrangement of intensive care unit (ICU) bed, blood and blood products from the blood bank, extra time taken to mobilize patients from COVID-19 suspect areas to the operation theater and donning of Personal Protective Equipment (PPE) by healthcare providers. 

The Government’s policy of curfew-like restrictions in hotspot areas, lockdowns with transportation hindrances leading to delays in arrival to the health facility, and fear of acquiring an infection during hospital visits may have resulted in an adverse effect on maternal and fetal outcomes [10,11]. Lack of awareness of danger signs, ignorance about a routine antenatal check-up, lack of performing routine investigations, antenatal ultrasound, and delayed admission at 41 weeks' gestation had resulted in missing out the severe complications like anemia, pregnancy-induced hypertension, PAS, and acute exacerbated bronchial asthma. The present case report highlights the impact of the COVID-19 pandemic on maternal mortality and stillbirths. The COVID-19 situation causes unprecedented delays in reaching the hospital, delays in receiving proper care, and suboptimal care leading to preventable maternal mortality and stillbirths.

## Conclusions

Therefore, in these pandemic times, the thought of COVID-19 always crosses the mind when we see a sudden worsening of clinical presentation in pregnancy. It might not be only due to COVID-19 but can also be due to other life-threatening conditions. It is also to be kept in mind that obstetric and antenatal care are essential services that should be continued, and people should be aware of it. This should be implemented by improving the structural competency of the healthcare workers to ensure the care of pregnant women with the support of Government and non-Governmental centers. Thereby, we can downplay the rising rate of maternal mortality and stillbirth.
